# Development of a tool for measuring uncertainty of acute cytotoxicity results using the methodology of OECD Guidance Document 129

**DOI:** 10.1016/j.namjnl.2025.100021

**Published:** 2025-04-14

**Authors:** Lorena de O. Neves, Bruno C. Garrido, José Mauro Granjeiro, Luciene B.L. Balottin

**Affiliations:** a Biology Metrology Division, Directory of Scientific Metrology, Industrial and Technology, National Institute of Metrology, Quality and Technology (INMETRO), Rio de Janeiro, Brazil; b Chemical Metrology Division, Directory of Scientific Metrology, Industrial and Technology, National Institute of Metrology, Quality and Technology (INMETRO), Rio de Janeiro, Brazil; c Directory of Scientific Metrology, Industrial and Technology, National Institute of Metrology, Quality and Technology (INMETRO), Rio de Janeiro, Brazil

**Keywords:** Non-animal methods, Uncertainty, Cytotoxicity, Reliability, Nams

## Abstract

Despite the use of strict acceptance criteria in validated methods for in vitro toxicology safety non-animal methods, the evaluation of statistical uncertainties in the results and their impact on the reproducibility and reliability of these methods is limited. Technical and operational variations in intra- and inter-laboratory settings can interfere with the results, and measurement uncertainty estimations are crucial for a better understanding of the potential sources of errors and ensuring that the assays are under control. The measurement uncertainty characterizes the dispersion of values, which can be attributed to the measurement results. This study applied the Monte Carlo statistical model to calculate the measurement uncertainty for the half-maximal inhibitory concentration (IC50) of sodium dodecyl sulfate (SDS) using OECD Guidance Document 129. We focused on acute oral cytotoxicity in vitro and developed a specific tool for this purpose. The Monte Carlo method enabled the identification of the contributions to the measurement uncertainty of IC50 and their confidence intervals for the in vitro cytotoxicity assay recommended by OECD GD 129. Furthermore, the statistical tool developed in this study, although specifically applied to OECD GD 129, has the potential to be adapted and extended to other in vitro toxicological assays based on dose-response curves, thus supporting broader applications in New Approach Methodologies (NAMs) by enhancing reproducibility and confidence in alternative methods validation processes. In conclusion, this study demonstrates the effectiveness of the Monte Carlo statistical model in quantifying the measurement uncertainty of sodium dodecyl sulfate (SDS) IC50 values, with a comprehensive analysis yielding a combined IC50 of 34.6 μg/mL with a combined uncertainty of 1.21 μg/mL. These findings highlight the importance of incorporating metrological evaluations to increase the reliability of in vitro methods. By enabling more informed decision-making, this study can significantly contribute to the advancement of toxicological research and NAMs.

## Highlights

• This study marks a significant metrological advancement in the OECD protocols.
• An open-access tool was developed for acute cytotoxicity uncertainty measurement.
• Measuring uncertainty of results can help the Chemical Safety Assessment.
• The combined IC50 and uncertainty values can be obtained, improving reliability.
• The integration of metrology can improve the reproducibility of non-animal methods.

## Introduction

Different animals, especially small ones, have been used in experiments because of their docility, practical handling, ease of reproduction, and short life cycles (Peter et al., 2017). However, such studies are not always justifiable, reproducible between species, or even proved to be irreplaceable (Knight, 2007; Ferdowsian and Beck, 2011). The rise of modern biomedical science has contributed to this scenario, e.i. to use computer tools and artificial intelligence (AI), while also influencing the growth of ideologies against the suffering caused to these animals (Wu et al., 2024).

Beginning in the 1970s, the Lethal Dose to 50 % of animals (LD50) test became widely used, becoming a prerequisite for the US Federal Food and Drug Administration (FDA) and being incorporated into the tests proposed by the Organization for Economic Cooperation and Development (OECD) in 1981, as guideline 401(5,6). At the same time, the OECD published similar acute dermal toxicity guidelines, making it possible to investigate the local and systemic effects.

After being incorporated by the OECD, the LD50 test now requires up to 100 animals to test each substance, with five males and five females per dose group, in three dose groups (with 5000 mg/kg being the maximum dose level), and the LD50 was estimated by observation of animals or historical data (Botham, 2002; United Nations. Acute toxicity. In: Globally Harmonized System of Classification & Labelling of Chemicals (GHS) 2007). In 1987, OECD 401 was revised mainly for animal welfare reasons, and the threshold dose was reduced to 2000 mg/kg. In 2000, as a result of two years of meetings with experts and raising awareness of the importance of the welfare of non-human animals, three alternatives were approved by the OECD peer review committee (guidelines 420, 423, and 425), reducing the number of animals required (between 8 and 15 per substance) and accepting that the animals were of only one sex ((United Nations. Acute toxicity. In: Globally Harmonized System of Classification & Labelling of Chemicals (GHS) 2007; Harthung, 2009)).

The consolidation of the relationship between in vitro cytotoxicity assays and the prediction of acute toxicity in vivo was published by the OECD in Guidance Document (GD) 129 in 2010. This document was based on the protocols recommended by the Interagency Coordinating Committee for the Validation of Alternative Methods (ICCVAM) and is the result of joint, independent validation studies organized by the National Toxicology Program (NTP) Interagency Center for the Evaluation of Alternative Toxicological Methods (NICEATM) and by the European Center for the Validation of Alternative Methods (ECVAM) (ICCVAM 2006; ICCVAM 2006, OECD 2014). In 2011, the European Union Reference Laboratory on Alternatives to Animal Testing (EURL ECVAM) overtook ECVAM activities (EC – European Commission 2010). The protocols described in GD 129, which use human cells, have the advantage of being an ethical strategy that spares the use of non-human animals.

Traditionally, the OECD GD 129 protocol has been used to estimate the initial doses for acute oral systemic toxicity tests, significantly reducing the number of animals used. This test, also called the basal cytotoxicity assay, delivers a half-maximal inhibitory concentration (IC50, that is, the concentration of a substance that induces death of 50 % of the cells when compared to untreated control cells), with which a cell viability curve is statistically drawn, correlating the concentration-effect (OECD 2014, Valadares, 2006). Given the possible intra- and inter-laboratory technical and operational variations that may interfere with the results, performing uncertainty calculations in chemical safety tests adds quality to measurements. Despite the acceptance requirements in the protocols established in OECD GD 129, there needs to be a discussion in the literature regarding the impact of statistical evaluation of results on the reproducibility and reliability of these analytical assays. Quantitative uncertainty assessments are critical not only for methodological rigor but also for regulatory toxicology, as they provide clearer confidence intervals that aid risk assessors and regulatory agencies in making informed decisions based on in vitro data.

Measurement uncertainty is the doubt about the true value that remains after performing a measurement. According to the Guide to the Expression of Uncertainty in Measurement (GUM), several causes can contribute to the uncertainty of a measurement, and they can be grouped into two main types according to the input data or extrapolations performed (JCGM 2012, Joint Committee for Guides in Metrology 106 J 2012). The measurement result is the set of values assigned to a measurand consisting of the measured value and associated measurement uncertainty.

The average IC50 values provided an estimate of the measured value. However, the measurement system can also be influenced by the dispersion of values around this average, including systematic errors or bias (associated with instruments, measurement techniques, and the experimenter) and random errors or standard deviation (unpredictable variations) (JCGM 2012). The trend and standard deviation are directly related to the veracity and precision of the data, which affects the total error and measurement uncertainty, respectively. Measurement errors affect repeatability, intermediate precision, and reproducibility (JCGM 2012, JCGM/WG 2008). This lack of quantified uncertainty is also a barrier to broader acceptance of in vitro methods and New Approach Methodologies (NAMs) in regulatory toxicology, where quantitative data with clearly defined confidence intervals are highly valued. Reporting values without knowledge of this associated dispersion is one cause of irreproducibility in science. Estimating the measurement uncertainty associated with inhibitory concentrations (IC50) is of the utmost importance to enable comparisons of values and informed decisions regarding these concentrations. In this study, we obtained the values of a control substance using a protocol validated by the OECD using murine cells.

Therefore, this study aimed to evaluate the performance of the OECD GD 129 by identifying its contributions to measurement uncertainty and creating a model to estimate this uncertainty using a Monte Carlo Simulation (MCS) method. Additionally, the ability to precisely quantify uncertainty contributes directly to the validation and regulatory acceptance of NAMs, promoting transparency and increasing reliability across laboratories and regulatory bodies. While this study specifically addresses the OECD GD 129 protocol, the statistical model developed herein can potentially be extended to other in vitro toxicological assays employing similar dose-response frameworks. Thus, our approach could serve as a generalizable framework for enhancing the reproducibility and reliability of various NAMs, supporting broader regulatory and scientific adoption.

## Methods

### Cell culture

BALB/c 3T3 clone A31 cells (Work Bank of the Laboratory of Tissue Bioengineering, Inmetro) were cultured in DMEM 4.5 g/L glucose with l-glutamine and sodium pyruvate (Lonza) supplemented with 10 % fetal bovine serum (FBS) (Gibco) and seeded at a concentration of 6.0 × 10^3^ cells/cm^2^ in a culture flask for adherent cells of 25 cm^2^ (SARSTEDT, cod. 83.3910002).

Except for SFB, the other reagents used to maintain BALB/c 3T3 clone A31 culture were free of animal products. The cells were cryopreserved in the animal origin-free medium. ProFreeze-CDM™ (Lonza) with 15 % (v/v) Dimethylsulfoxide/DMSO (Sigma-Aldrich), obtaining viability > 99.0 % on thawing. Half of the culture medium was replaced twice a week until the culture reached approximately 60 % confluence. At that point, fibroblasts underwent enzymatic dissociation with an animal origin-free solution, TrypLE™ Express (Gibco), being the recombinant enzyme neutralized with the complete culture medium.

### Neutral red uptake (NRU) assay

To perform the NRU 3T3 assay, the cells were cultured at appropriate concentrations for incubation periods of two days, three or four days, depending on the routine. This was necessary so that the cultures did not exceed 70 % – 80 % confluence, avoiding contact inhibition. The cells were subcultured when they reached high cell confluency (approximately 80 %). The BALB/c 3T3 clone A31 line was seeded in a 96-well plate with fresh plating medium containing DMEM High (Lonza) 10 % FBS, 1X Penicillin-Streptomycin [100 IU/ mL], and 4 mM l-glutamine at a concentration of 2.5 × 10^3^ cells/well, and incubated in an oven (37 °*C* ± 1 °C, 90 % ± 10 % humidity, 5.0 % ± 1.0 % CO_2_ /air) for 24 ± 2 h to reach 50 % confluence.

Sodium dodecyl sulfate (SDS) (CAS: 151–21–3, Sigma-Aldrich) was used as the positive control (CP), as recommended by OECD GD 129. The plating solution was removed, and in the wells with cells designated from C1 to C8, an additional 50 μL of DMEM High dilution solution, 2X Penicillin-Streptomycin [100 IU/mL], and 4 mM l-glutamine were applied, with the respective SDS dilutions. After 48 h, the solutions were removed from the wells and washed with Dulbecco's phosphate-buffered saline (DPBS) for the NRU assay. For the Main Assay with CP, 10 mg/mL SDS stock solutions (Sigma-Aldrich) were used to obtain a C1 concentration of 100 μg/mL after a 100X dilution. Blank wells can be filled with Dulbecco's phosphate-buffered saline (PBS/DPBS), as done here, or with the routine medium. The culture was incubated (37 °*C* ± 1 °C, 90 % ± 10 % humidity, 5.0 % ± 1.0 % CO_2_ /air) for 48 ± 0.5 h.

After approximately 46 h, the plates with the culture were examined under a light microscope to identify and record any plating errors, contamination, or other irregularities. A neutral red dye stock solution (25 μg/mL for BALB/c 3T3 and 33 μg/mL for NHK) was prepared and kept overnight in an incubator (37 °*C* ± 1 °C, 90 % ± 10 % humidity, 5.0 % ± 1.0 % CO_2_ /air).

In the NRU 3T3 assay, the NR dye stock solution (Sigma-Aldrich) was diluted in a DMEM High solution with 5 % FBS, 1X Penicillin-Streptomycin [100 IU/mL], and 4 mM l-glutamine. All plate wells were carefully rinsed with 250 μL/well of pre-warmed PBS, inverted, and dried with sterile absorbent paper. Then, 250 μL of VN solution was added to each well (including the blank) for incubation (37 °*C* ± 1 C, 90 % ± 10 % humidity, 5.0 % ± 1.0 % CO_2_ /air) for 3.0 ± 0.1 h.

After incubation with the NR dye solution, the plate was rinsed with 250 μL/well of PBS (Lonza) and exposed to 100 μL/well of desorbing solution (water: ethanol: acetic acid; 49:50:1, v/v/v), water, ethanol (VETEC, 99.8 % pure, batch: DCBC7468V), and glacial acetic acid (Sigma-Aldrich, ≥ 99.0 % pure), 49:50:1 (v/v/v). The mixture was shaken for 20–45 min, stopped for 5 min, and analyzed using a spectrophotometer at an optical density of 540 nm ± 10 nm (OD_540_). The results were applied to a linear regression calculation using the values of IC50 and LD50 orally in rodents: Log LD50 (mg/kg) = 0.372 × log IC50 (µg/mL) + 2.024 (OECD 2014).

### Measurement uncertainty

The estimation of the measurement uncertainty was initiated by identifying the main input quantities influencing the measurement results and quantifying their uncertainties. The estimated value for the measurand, the sensitivity coefficients of each quantity, the combined standard uncertainty of the measurand (u_c_), and the effective degrees of freedom *(v_eff_*) of the combined standard uncertainty were calculated (Zakharov et al., 2021). A minimum of four replicates is recommended to maintain confidence in uncertainty estimations, while ten or more replicates allow for conventional use of a coverage factor *k* = 2, assuming a normal distribution. The effective degrees of freedom decrease when fewer replicates are used, leading to increased uncertainty, as described in ISO 8655–10:2022, section 6.4.2 (Joint Committee for Guides in Metrology 106 J 2012).

Type B uncertainties were obtained from technical sources such as calibration certificates or reference data. Type A uncertainties are determined when there is statistical information; for example, they can be calculated from the mean of repeated measurements (*x*), calculating the experimental standard deviation (s), based on Equation 1, where the number of repetitions (*n*) was and should be at least 10. The size of the output uncertainty depends directly on the input uncertainties.1

Volumetric instruments such as graduated pipettes, unlike electronic micropipettes or manual pipettors, are not precisely calibrated, and the scale serves only as an approximation; therefore, assessing the uncertainty associated with their use is necessary. According to the ISO 8655:2022 series, both systematic and random uncertainties affect pipetting accuracy, with their magnitude dependent on the number of replicate measurements (International Organization for Standardization 2024). Based on the calibration certificate of the multichannel micropipette, the maximum permissible systematic error (accuracy) was ±1 %, while the maximum permissible random error (precision) was ≤ 0.4 %.

The sensitivity coefficients (*s*) are partial derivatives *∂f / ∂xi*, which are equivalent to *∂f / ∂X_i_* calculated at *X_i_ = x_i_*, describing how the output quantities (*Y*) vary with changes in the inputs (*x_1,_ x_2,_ x_3,_… x_n)_* . Since *Y = f(X_1,_ X_2,_ X_3,_… X_n_),* the combined uncertainty (*u_c_*) is the sum of the estimated variance for *Y* generated by the estimated variance associated with each input quantity *x_i_* that is, *∆Y = ∂f / ∂x_i_ ‘ ∆x_i_* . Using this, the uncertainty was calculated according to Equation 2.

The ambient conditions of the method were recorded as follows: ambient temperature 21.77 °C, relative humidity 60.55 %, atmospheric pressure 1017.07 hPa, and distilled water temperature 21.1 °C. The volume was obtained using the function in Equation 3.

Where: *V*_20_ = volume, at a temperature of 20 °C (mL); *m*= (I_L_ -I_E_) the net balance reading (g); where I_L_ is the result of weighing the balance after pipetting, I_E_ is the result of weighing before pipetting; *ρ_m_*= reference density of weights used to adjust balance (i.e. 8 g/mL); *ρ_a_* = air density, in grams per milliliter, g/ml; *∝_t_* = coefficient of thermal expansion of the pipette (L/ °C; *t_dW_* = temperature of the distilled water ( °C).

For the gravimetric calculations involving the pipettes, the equations recommended by ISO 4787:2010 (Calibration Guide n° 19 of the European Association of National Metrology Institutes) and DOQ-CGCRE-027 (Guidance for the Accreditation of Laboratories in the volume area) were used (EURAMET e.V 2018, INMETRO 2011).

For volume gravimetry calculations, the density of pure water at the calculation temperature t °C, in g/mL, (*ρ_W_*), used as the test liquid, was determined using the Tanaka formula (EURAMET e.V 2018), given by Equation (4). Where: *t* = water temperature ( °C); *a_1_* = -3.983035 °C; *a_2_* = 301.797 °C; *a_3_* = 522,528.9 ( °C)^2^; *a_4_* = 69.34881 °C; *a_5_* = 0.999974950 g/ml.

To calculate the standard uncertainty regarding the water temperature, the value given by Tanaka = 9 × 10^–7^ g/mL was used, with *k* = 2 and the uncertainty of the thermometer calibration given by the certificate. The formula used to calculate the expanded uncertainty of the water density is given in Equation 5.

The standard uncertainty of the specific air mass also needs to be considered, as the instruments that displaced the internal air for liquid aspiration were evaluated. To define u(ρ_A_), the ρ_A_ and U(ρ_A_) values were calculated from Equations 6 and 7, respectively.

Where: *k*_1_ = 3,4844 × 10^–1^ °C.kg/(mL.hPa); *k*_2_ = 2.52 × 10^–3^ kg.g/ml; *k*_3_ = 2.0582 × 10^–5^ °C.kg/ mL; *k*_4_ = 273.15 °C; $\bar{\rho_{A\;}}$= Mean Atmospheric Pressure, in hPa; $\bar{\text{UR}}$= Average Relative Air Humidity, in %; $\bar{T_{A}}$ = Average ambient temperature, in °C.

Where: *bar_p_ =* Pressure on the Barometer Calibration Certificate, in hPa; *terh_T_ =* Temperature *in the* Thermohygrometer Calibration Certificate, in °C; *terh_U_ =* Humidity in the Thermohygrometer Calibration Certificate, in % RH; *s* = Sensitivity coefficient.

The sensitivity coefficients for each input quantity were calculated from the terms in Equations 8, 9, 10, and 11.

After all parameters related to the volume in the sample wells and concentrations of test solutions were estimated, and the repeatability between different sample wells, the final combined uncertainty associated with each concentration value was estimated. Uncertainties associated with each viability report were also estimated, and these estimates were used to build normal distributions that fed the Hill regression curves into a Monte Carlo Simulation (MCS). As noted by Fuentes-García and collaborators, there is lack of consensus in the literature about the ideal number of Monte Carlo Simulations to be run and several considerations have to be taken into account, including computation time. The authors report 1500 simulations to be sufficient to achieve reliable uncertainty estimates. We adopted 10,000 simulations in our work (Fuentes-García et al., 2024).

The greater the number of simulations, the better are the calculated results. To improve the efficiency of the calculations and enable the adoption of the MCS method by other laboratories, a web-based application was developed by us, called HillGD129 v1.1 (Garrido, 2023), using ‘shiny’ (Chang et al., 2023) was built to perform the calculations. This application was built using R version 4.2.1 (R Core Team 2022), and the packages dplyr (Wickham et al., 2023), Rhandsontable (Owen et al., 2021), bslib (Sievert and Cheng, 2022), and drc (Ritz et al., 2015). Reporting functionalities were added to the application using R markdown (Allaire et al., 2022, Xie et al., 2018, Xie et al., 2020) and knitr (Xie, 2014, Xie, 2022) which estimates the measurement uncertainties by using all uncertainties related to the input quantities in both axes of the fitted Hill equation and then performing MCS. This leads to a large set of >1000 IC50 results, the distribution of which is used to estimate the measurement uncertainty of IC50.

We utilized the NIST Consensus Builder (A Koepke et al., 2017) according to the recommendations provided in the application HillGD129 v1.1. The Hierarchical Bayes (Laplace) method was applied to combine the results from multiple sources, ensuring robust and statistically sound estimation. This approach enables better uncertainty quantification by integrating prior distributions with experimental data, thereby yielding more accurate results (Kwon et al., 2020). The Consensus Builder facilitates the process by offering a systematic framework for combining data and generating consensus results based on Bayesian principles (A Koepke et al., 2017).

## Results

### NRU test implementation – OECD GD 129

**Fig 1:**
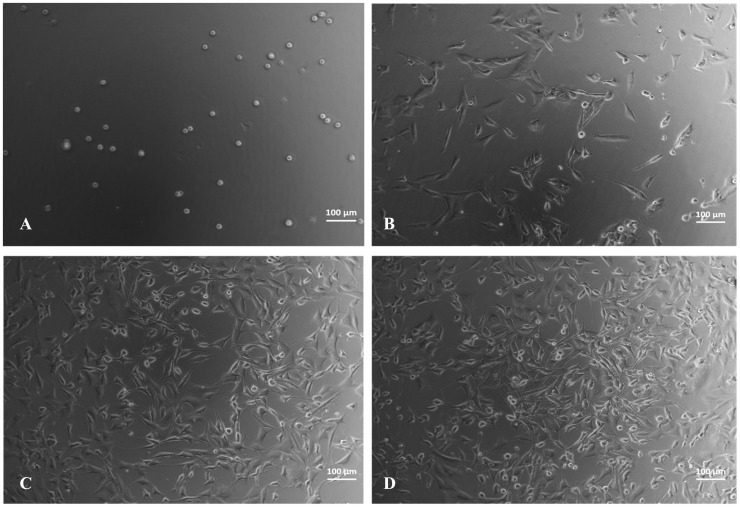
Micrographs of the wells representing the Vehicle Control. BALB/c 3T3 clone A31 incubated at 37 °*C* ± 1 °C, 90 % ± 10 % humidity, 5.0 % ± 1.0 % CO_2_/air. A – After plating 2.5 x 10^3^ cells in 100 μ*L*. B – After incubation of 24 *h* ± 2 h. C – Left Vehicle Control (VC1) after exposure for 46 h. D – Right Vehicle Control (VC2) after exposure for 46 h.

BALB c/ 3T3 clone A31 showed conformal morphology (Fig. 1), ensuring a reliable cytotoxicity assessment. In addition to verifying uniform cell seeding, consistent growth patterns, and absence of crystal formation, we obtained an adequate mean CV, with variability between replicates not exceeding 15 %. These measures ensured that the calculated IC50 values were not influenced by systematic errors or inconsistencies in cell viability measurements, with values reaching around the expected typical dose-response curve for SDS in the NRU 3T3 assay.

**Fig 2:**
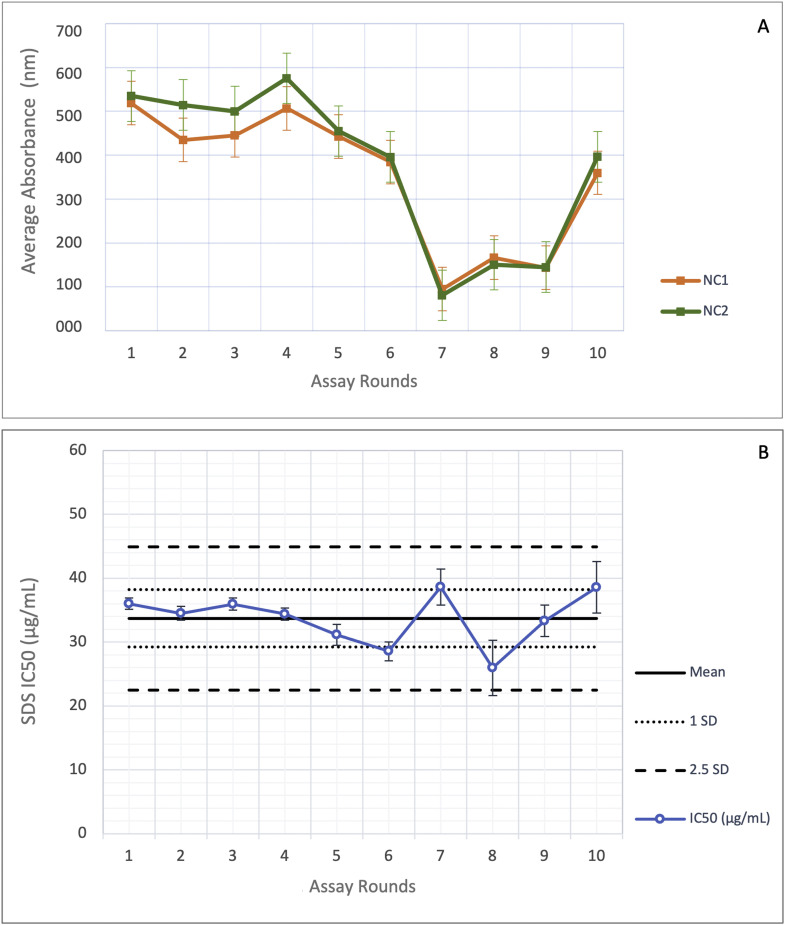
Historical series in NRU 3T3 assays. The historical series comprises the results obtained in the laboratory, seven retrospective results, and the results of the present study. Mean absorbance values of negative controls (cells on vehicle) across multiple experimental replicates (A). This control chart monitors the stability of the negative control responses, ensuring consistency across assays. The data represent the average absorbance values with standard deviations, subsequent to blank correction. IC50 values for sodium dodecyl sulfate (SDS) obtained from multiple independent rounds of the NRU 3T3 assay (B). IC50 equivalent to (1) 36.02 µg/mL; (2) 34.51 µg/mL; (3) 35.95 µg/mL; (4) 34.39 µg/mL; (5) 31.16 µg/mL; (6) 28.58 µg/mL; (7) 38.61 µg/mL; (8) 25.96 µg/mL; (9) 33.32 µg/mL; (10) 38.58 µg/mL. Limits: mean of 33.71 µg/mL; Standard deviation (SD) of 4.49 µg/mL and 2.5 SD with a value of 11.23 µg/mL.

A control chart was constructed using the absorbance values obtained from the vehicle control readings, following blank correction. This approach enables the monitoring of the stability of negative control responses across experiments, aiding in the detection of potential systematic variations (Fig. 2.A).

The protocol in the OECD's GD 129 indicates that the mean value of the expected IC50 for the SDS is 41.5 ± 4.8 *μ*g/ mL for the NRU assay with BALB c/ 3T3, based on 233 experiments. However, the SDS IC50 was within ± 2.5 SD of the historical average established in the laboratory control chart, as recommended by GD 129, validating the implementation of the method. Based on a historical series (10 experiments), the mean IC50 was 33.71 ± 4.49 μg/mL, calculated using GraphPad Prism® 6 statistical software (v. 6.01) (Fig. 2.B).

Linear regression calculations revealed that the test runs in the present work that met the acceptance criteria also correctly proved that SDS belongs to Category 4 of the UN GHS in the classification of acute oral toxicity.

### Calculation of measurement uncertainty of the method

**Fig 3:**
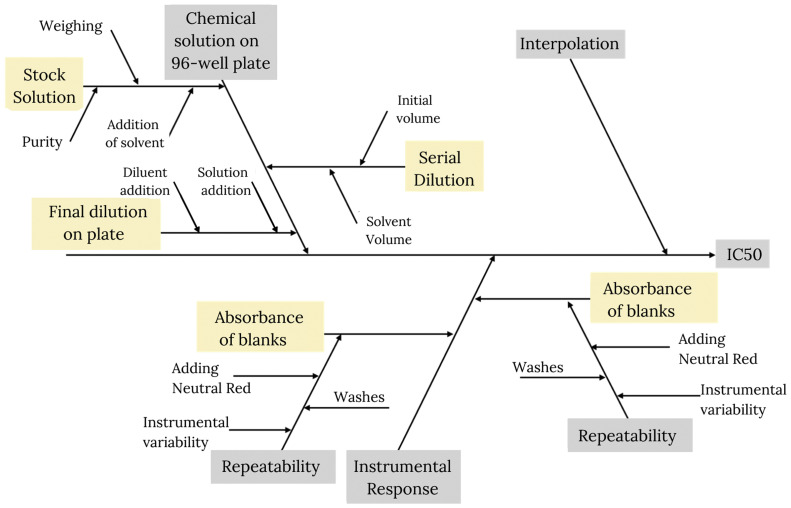
Cause-and-effect Ishikawa diagram in the Neutral Red Uptake (NRU) test of OECD GD 129. The sources of uncertainty linked to the test were identified to estimate the measurement uncertainty associated with the traditional protocol.

The method recommended by GD 129 with BALB/c 3T3 cells (3T3 NRU) presents the sources and contributions to the measurement uncertainty, identified, and organized in an Ishikawa diagram (Fig. 3). The inclusion of uncertainty sources in the diagram was based on their measurability and direct influence on the analytical steps of the NRU assay. Only sources that could be traced to quantifiable parameters (e.g., pipetting accuracy, temperature, density) were included in the numerical model (Table 1). Though acknowledged as relevant, elements such as plate reader performance and biological variability were not isolated as independent contributors due to the difficulty of direct quantification. Instead, their effects are encompassed within the overall repeatability term derived from experimental replicates. This strategy aligns with recommendations in ISO 21,748:2017 and GUM-S1 for empirical models, where non-separable uncertainty contributions may be grouped under repeatability or reproducibility components.

**Table 1:** Uncertainty numerical contributions of the method in OECD GD 129. These are the contributions to the final IC50 uncertainty value, which must be included numerically in the template spreadsheet to estimate the uncertainties of the assay concentrations and responses on HillGD129 v1.1.

	Dilutions Steps
Sources	Initial Volume (µL)
	Expanded Pipetting Uncertainty (µL)
	Coverage Factor (k)
	V half (mL)
	Expanded Pipetting Uncertainty (mL)
	Coverage Factor (k)
	Concentration (µg/mL)
	Concentration Uncertainty (µg/mL)
	Chemical's Volume Pipetted per Well (µL)
	Expanded Pipetting Uncertainty (µL)
	Coverage Factor (k)
	Dilution volume pipetted into the well (µL)
	Expanded Pipetting Uncertainty (µL)
	Coverage Factor (k)
	Concentration (µg/mL)
	Concentration Uncertainty (µg/mL)

**Fig 4:**
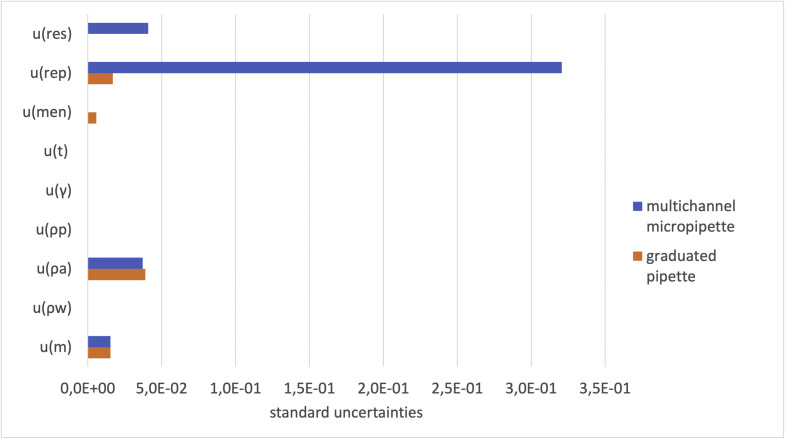
Contribution of the standard uncertainties of the volume measured using the Multichannel Micropipette and Graduated Pipette. These are standard uncertainty values ​​on the x-axis. To Multichannel Micropipette the expanded air density uncertainty and standard deviation of the measurements are the significant contributors. To Graduated Pipette the expanded air density uncertainty and standard deviation of measurements are the significant contributors.

The calculations showed that the source of the most significant contribution to estimating the combined uncertainty of the volumes removed by the graduated pipette was the uncertainty of the specific mass of air. At the same time, repeatability had the greatest contribution in estimating the combined uncertainty of the volumes removed by the multichannel micropipette (Fig. 4). The sources contributing to the expanded uncertainty of the volume removed with the Multichannel Micropipette and Graduated Pipette are listed in Tables A.1 and A.2.

The IC50 values and their associated uncertainties were determined by applying the MCS. The expansion factor (k) was selected based on the confidence interval, and the expanded uncertainty (U) was determined using the function *U* = *k* × uc.

All the uncertainties presented in Table 1 are the uncertainty contributions of the method in OECD GD 129, which were also incorporated into the Monte Carlo Method. Following the GD 129 cytotoxicity assay with BALB/c 3T3 clone A31 cells, the IC50 of SDS was calculated using the HillGD129 v1.1 software with 10,000 Monte Carlo simulations (Sievert and Cheng, 2022). MCS for the rounds using the web-based application with simulations resulted in the values shown in Table 2.

**Table 2:** Results of multiple data of IC50 in dose-response curve for SDS historical series in NRU 3T3 assays.

Rounds	IC50	Standard_unc	Expanded_unc	Hill	min_value	max_value	ec_50
1	36.02	0.87	1.74	11.46	0.20	96.49	36.24
2	34.51	1.08	2.16	12.54	-0.35	107.47	34.15
3	35.95	0.94	1.88	9.99	-0.25	103.89	35.70
4	34.39	0.96	1.93	9.20	-1.26	102.25	34.32
5	31.16	1.65	3.30	6.97	-1.52	96.15	31.65
6	28.58	1.49	2.98	10.54	0.20	87.63	29.35
7	38.61	2.78	5.56	14.76	3.55	110.21	37.94
8	25.96	4.30	8.59	39.89	23.07	56.01	26.96
9	33.32	2.43	4.87	3.97	-10.87	65.86	46.75
10	47.56	4.35	8.70	3.18	-0.10	92.51	50.08

**Fig 5:**
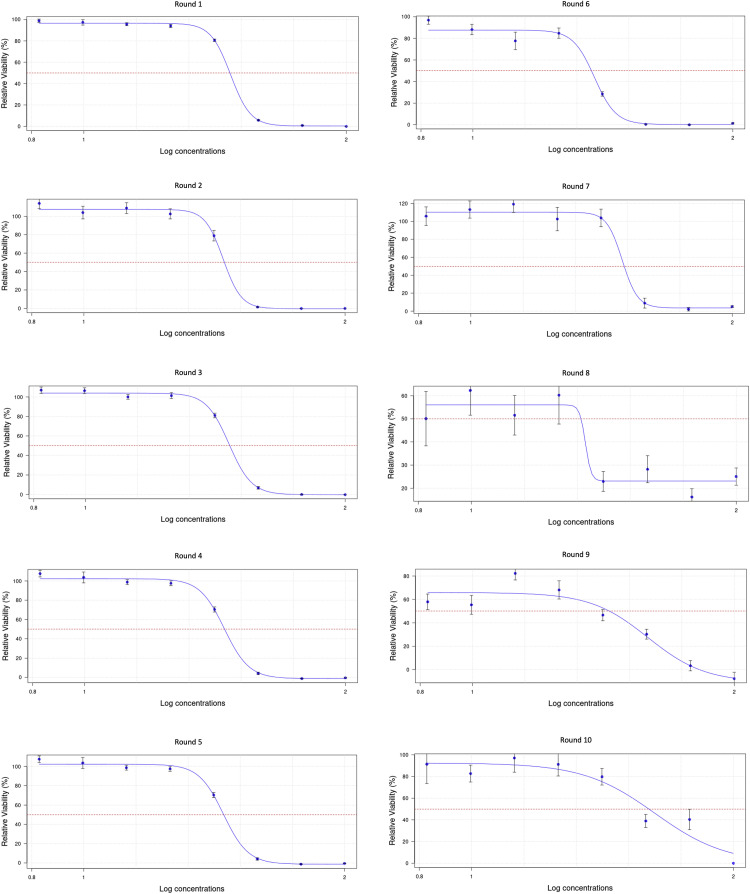
Graphical results of the cytotoxicity analyses. Dose-response curve for sodium dodecyl sulfate (SDS) in the NRU 3T3 assay. The dots show the mean relative viability ( %) of the six replicates produced at each of the eight chemical concentrations.

**Fig 6:**
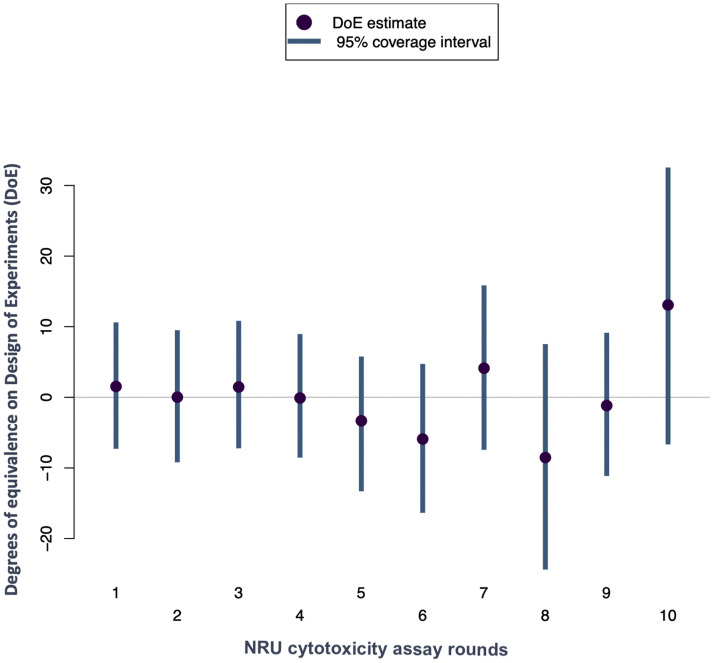
Unilateral degrees of equivalence on Design of Experiments (DoE) based on Bayesian procedures. The purple points indicate the Bayesian posterior means for each evaluated sample, and the blue error bars reflect the corresponding uncertainty (95 % credible intervals). These intervals represent the range within which the true value of the degree of equivalence is expected to fall with 95 % probability. Any points significantly deviating from zero suggest a systematic bias, while values close to zero indicate agreement with the consensus value within the uncertainty limits.

The graphical representation of the dose-response curves is shown in Fig. 5. Using the Hierarchical Bayes (Laplace) method, the combined IC50 for the SDS was 34.6 μg/mL with a combined uncertainty of 1.21 μg/mL (Fig. 6). This level of uncertainty represents a relative variation of approximately 3.5 % in relation to the combined IC50 value. In practical terms, this narrow confidence range provides robust support for decision-making processes in toxicological classification, particularly when threshold values are used to determine GHS categories or regulatory cut-offs. In a regulatory context, such precision is essential for ensuring the reproducibility of classifications across laboratories and for reducing false positives or negatives in hazard identification.

## Discussion

According to OECD GD 129, a historical series must be constructed in the laboratory, including all IC50 values obtained for CP, to implement the cytotoxicity assay. The document recommends a control chart of at least ten tests for the CP used in the NRU, where the IC50 must be within ± 2.5 SD of the historical average established in the laboratory for the tests to be accepted.

Other parameters must be considered, such as sufficient cytotoxicity to calculate the IC50, the absence of NR crystals and a good fit of the concentration-response data for the HillSlope function. To achieve the latter, it is necessary to comply with the acceptance criteria of Blank General Average of ∼0.05, Vehicle Control average equal to 0.476 ± 0.117, difference ≤ 15 % between the means of VC1 and VC2 corrected with the blank, cell seeding control; correlation coefficient (R^2^) ≥ 0.85 %.

Although cell density has been identified as a factor that may influence cell viability assays, no further investigations were conducted in this study, as the same cell density was strictly standardized and inoculated per well in all experimental replicates (Gasque et al., 2014). Additionally, only relative cell viability is considered in this assay, which minimizes the potential impact of absolute cell density on the results. However, future studies could further explore the relationship between cell density and assay variability, providing a more comprehensive understanding of this factor as a potential source of measurement uncertainty.

Typically, the reading values at OD_540_ for SDS should not be less than 0.3; however, measurements below this value may be justified if the cells are shown to be healthy and the toxicity response is adequate.

### Measurement uncertainties and strategies for reduction

According to the Guide to the Expression of Uncertainty in Measurement (GUM), measurement quality depends on the sum of the technical uncertainties involved and therefore includes pre-analytical, analytical, and post-analytical uncertainties (Joint Committee for Guides in Metrology 106 J 2012). Therefore, performing an adequate statistical assessment to identify, characterize, and reduce measurement uncertainty can increase the relevance, reliability, and completeness of the data obtained by the method, which is an important element for evidence-based decision-making (OECD 2020). Pre-analytical uncertainties arise from factors related to test inputs and involve stability, transportation, storage, and preparation of materials, which can interfere with the relevance, reliability, and completeness of the data obtained. Post-analytical steps include interpretation, integration of phenomena, and extrapolation of results (González et al., 2018). Analytical measurement uncertainty is associated with factors such as equipment calibration, concentrations, composition and variability between batches of reagents and samples, operator performance, external interferences, and the intrinsic uncertainty of the measurand. Having clarity about what these are and the acceptable values for each of the analytical factors, when quantifiable, uncertainty estimation is useful for mapping gaps in the method and indicating the quality and confidence level of the results (MATSUDA et al., 2015). In the present study, identifying the sources that contribute to uncertainties within OECD GD 129, presented in the Ishikawa Diagram, was essential for outlining subsequent calculations.

Using the methodology proposed in OECD GD 129 for cytotoxicity testing by NRU uptake, it was not possible to quantitatively assess the impact of the technical operator's performance on the calculation of uncertainty. The minimum requirement necessary to perform the test covered by OECD GD 129 is knowledge of the Guidance Document on Good In Vitro Method Practices (GIVIMP) and Good Laboratory Practices (GLP) principles. However, its impact may be indirect in the uncertainties associated with pipetting. As seen from the results, the expanded uncertainty values for the SDS IC50 ranged from 0.87 to 4.31 µg/mL, showing that different operators, at different times, and with different equipment can impact the uncertainty of the final reading.

The results (Fig. 3) show that the primary contributors to the uncertainty in both the multichannel micropipette and graduated pipette measurements are the air density uncertainty u(pa) and the repeatability of the measurements u(rep). Specifically, the air density u(pa) is a crucial factor in calibration procedures, as variations in environmental conditions such as temperature, humidity, and atmospheric pressure significantly affect air density measurement. It is particularly challenging to measure accurately in such protocols, contributing to the slightly higher uncertainty values observed (ML et al., 2021, Szymiczek, 2020).

For the multichannel micropipette, the repeatability of measurements u(rep) is the most dominant factor contributing to the uncertainty, with a substantially larger impact than other factors. This is likely due to the inherent difficulty of achieving consistent liquid handling across multiple channels. Additionally, the air density uncertainty is significant, reflecting the sensitivity of the system to ambient conditions, which is challenging to control.

Air bubbles in microplate wells can introduce measurement bias in plate reader assays by affecting light absorption and scattering (Petersen et al., 2022). However, in the present study, this uncertainty was not included in the IC50 calculations, because we implemented specific control measures to mitigate bubble formation. One of the key strategies used was reverse pipetting with multichannel micropipette, a technique that reduces the introduction of air bubbles by ensuring consistent liquid dispensing and avoiding splashing. Reverse pipetting has been recognized as an effective method for enhancing the precision of liquid handling (Pandya et al., 2010). Additionally, visual inspection of the wells was performed before measurements, and when necessary, sterile needles were used to remove any remaining air bubbles, further reducing the potential bias. These procedural controls are aligned with the best practices recommended by Petersen et al. (2022) to improve the reliability of microplate-based assays.

In our study, we acknowledged that air bubbles would influence the measurement of IC50 values; however, this factor was not explicitly quantified. Future studies could evaluate the degree of measurement uncertainty introduced by air bubbles and establish quantitative corrections if necessary. We incentivize the assessment of the robustness of different mitigation strategies.

In contrast, for the graduated pipette, the contribution of air density uncertainty u(pa) is nearly as impactful as that for the multichannel micropipette, but the repeatability u(rep) is comparatively smaller. This suggests that while environmental factors play a major role in the uncertainty for both types of pipettes, the graduated pipette offers more consistent handling, likely because of its simpler design and operation compared to the multichannel micropipette.

Addressing the uncertainty in the air density, which is difficult to measure accurately, is crucial for reducing the overall uncertainty in both pipetting methods. However, we do not dwell on this discussion in this study. This can be achieved through stricter control of environmental conditions during calibration (Batista et al., 2007). Moreover, improving the repeatability of multichannel micropipettes can significantly reduce uncertainties, further enhancing the precision of volume measurements in experimental setups. In this case, pipetting automation may be helpful.

### Monte carlo simulation application developed for OECD GD 129

Our novel web-based application led to a robust estimation of the measurement uncertainty of the procedure with a convenient and user-friendly interface. This means that the approach presented in this study can be used in several other fields, provided that the underlying model is the Hill equation. It is important to highlight that we applied a comprehensive measurement uncertainty estimation using all the input quantities and numerical simulations to estimate the Hill curve parameters, which has never been reported before. This improvement adds reliability to the values measured using OECD GD 129. Moreover, the methodology employed here—combining input uncertainty modeling, Monte Carlo simulation, and Bayesian consensus—can be adapted to other in vitro models that rely on dose-response analysis, such as those used for endocrine disruption, skin sensitization, or neurotoxicity. Applying this framework more broadly can enhance transparency and reproducibility across diverse NAMs, accelerating their validation and regulatory acceptance.

The initial concept of the GD 129 was to provide a structured approach for estimating the starting dose for in vivo testing (OECD 2014). However, its application has expanded significantly, and it is now widely used as an initial screening tool in numerous studies. In cases where the estimated initial dose exceeds 2000 mg/kg body weight, further in vivo testing may not be required, thereby contributing to the reduction and refinement of animal testing in line with 3Rs principles. This broader applicability underscores the relevance of in vitro approaches to regulatory toxicology and risk assessments (EURL ECVAM EURL for A to AT 2025).

By improving the reliability of dose estimations, the implementation of robust measurement uncertainty assessments, such as those enabled by the Monte Carlo method, further enhances confidence in these in vitro methods, supporting their integration into NAMs. Future research should explore additional in vitro applications of OECD GD 129 in various toxicological models, reinforcing its role beyond dose selection and expanding its impact on alternative testing strategies.

The 2nd edition of the Guidance Document on evaluating and expressing uncertainty in hazard characterization stresses the necessity for a clear and thorough examination of uncertainties during the assessment process. This highlights that transparency and systematic evaluations are key to ensuring the reliability of the conclusions drawn from hazard characterization (WHO/IPCS 2018).

This represents a landmark for comparing results in this field because measurement uncertainty estimates are essential for comparing results within and between laboratories. By quantifying measurement uncertainty, informed decision-making based on collected data is facilitated. The web-based software developed in this study will prove invaluable not only in assessing intra-assay uncertainties but also in evaluating inter-assay or inter-laboratory reproducibility. This application is available for free (Garrido, 2023) and can be used by researchers and technicians when following the GD 129 protocol as a tool for building the required curves and calculating their associated uncertainties.

A simple combination of these results by pooling was not appropriate in this case to describe the distribution of values due to the presence of extreme results which influence greatly the measurement uncertainty estimate. Therefore, we applied a Random Effects Model using the Hierarchical Bayes method with Laplace using the NIST Consensus Builder to achieve a robust combination of the results while taking into account their individual measurement uncertainties.

This study supports integrating metrological principles into toxicological risk assessment by framing uncertainty as a quantifiable and traceable element of the experimental process. Such approaches align with recent international guidelines that call for more rigorous uncertainty characterization in hazard identification and classification. Significantly, this work contributes to the broader effort of standardizing in vitro test interpretation and improving comparability across laboratories, essential for consolidating NAMs in regulatory science.

## Conclusions

In conclusion, our study effectively demonstrates the utility of the Monte Carlo Simulation method for accurately estimating the measurement uncertainty of IC50 values in cytotoxicity assays, in compliance with OECD Guidance Document 129. We have successfully quantified the statistical uncertainties affecting IC50 measurements for SDS, with a comprehensive analysis yielding a combined IC50 of 34.6 μg/mL with a combined uncertainty of 1.21 μg/mL, thereby enhancing the reliability and reproducibility of these assays. The development of a web-based application for uncertainty estimation represents a significant advancement in facilitating better decision-making in toxicological research and safety assessments. Although this work focused on the OECD GD 129 protocol and SDS as a test substance, the integrated approach—comprising measurement uncertainty estimation, Monte Carlo simulation, and consensus modeling—can be adapted to other dose-response-based in vitro methods. This flexibility strengthens the validation process of alternative assays and supports broader regulatory acceptance of NAMs through improved reproducibility, transparency, and traceability. Furthermore, the availability of a freely accessible web-based application for estimating measurement uncertainty encourages the adoption of best practices in laboratories worldwide, thereby fostering standardization and harmonization across the scientific community. This study underscores the critical role of rigorous repeatability evaluations in improving the precision of toxicological safety tests, incorporating metrological evaluations to increase the reliability of in vitro methods.

## CRediT authorship contribution statement

**Lorena de O. Neves:** Writing – review & editing, Writing – original draft, Visualization, Validation, Resources, Methodology, Investigation, Formal analysis, Data curation, Conceptualization. **Bruno C. Garrido:** Writing – original draft, Software, Formal analysis, Data curation, Conceptualization. **José Mauro Granjeiro:** Writing – original draft, Validation, Supervision, Funding acquisition. **Luciene B.L. Balottin:** Supervision, Resources, Project administration, Funding acquisition, Data curation, Conceptualization.

## Declaration of competing interest

The authors declare that they have no known competing financial interests or personal relationships that could have appeared to influence the work reported in this paper.

## Data Availability

Data will be made available on request.

## Appendix Supplementary materials

Image, application 1

Image, application 2
