# Supplementary material for: Development of a tool for measuring uncertainty of acute cytotoxicity results using the methodology of OECD Guidance Document 129
Source: NAM J. 2025 Apr 14;1:100021. doi: 10.1016/j.namjnl.2025.100021 (PMC13396913; doi:10.1016/j.namjnl.2025.100021)
Supplement: Supplementary file 1 [file mmc1.docx]

| **Sources** | **Contributions** | **Type** | **Standard Uncertainty** | **Sensitivity coefficient** |
| --- | --- | --- | --- | --- |
| **Mass** | Scale Certificate | B | 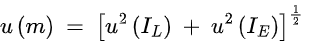  (1) | 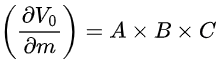  (2) |
|  | Balance Resolution | B |  |  |
|  | Evaporated mass | B |  |  |
| **Specific mass of water** | Water Control | B | 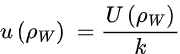(3) | 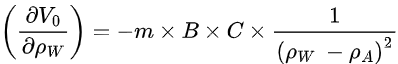  (4) |
| **Specific air mass** | Specific air mass | B | 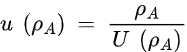  (5) | 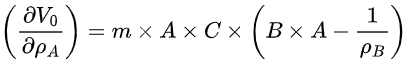  (6) |
| **Specific mass of reference weight** | Estimated value | B | 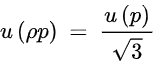  (7) | 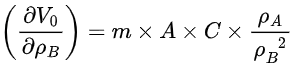  (8) |
| **Material thermal expansion coefficient** | Estimated value | B | 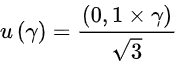  (9) | 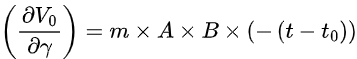  (10) |
| **Liquid Temperature** | Thermometer calibration certificate | B | 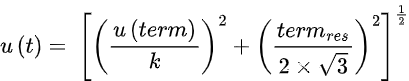  (11) | 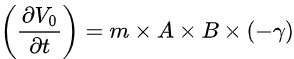  (12) |
|  | Thermometer Resolution | B |  |  |
| **Repeatability** | Standard deviation referring to the calculated volume | A | 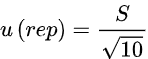  (13) | 1 |
| **Micropipette Resolution** | Calibrated instrument resolution | B | 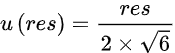  (14) | 1 |

**Table A.1. Sources that contribute to the expanded uncertainty of the volume removed with the Multichannel Micropipette.** Uncertainty factors: Type A – obtained in the test under repeatability conditions; Type B – obtained from available technical sources.

| **Sources** | **Contributions** | **Type** | **Standard Uncertainty** | **Sensitivity coefficient** |
| --- | --- | --- | --- | --- |
| **Mass** | Scale Certificate | B | 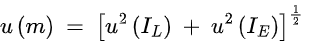 | 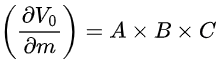 |
|  | Balance Resolution | B |  |  |
|  | Evaporated mass | B |  |  |
| **Specific mass of water** | Water Control | B | 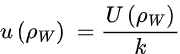 | 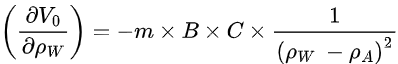 |
| **Specific air mass** | Specific air mass | B | 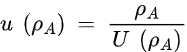 | 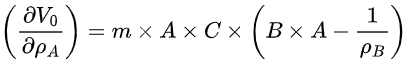 |
| **Specific mass of reference weight** | Estimated value | B | 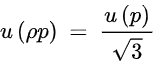 | 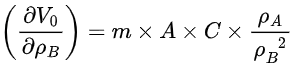 |
| **Material thermal expansion coefficient** | Estimated value | B | 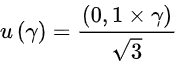 | 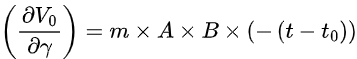 |
| **Liquid Temperature** | Thermometer calibration certificate | B | 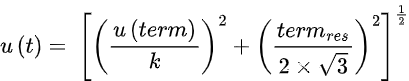 | 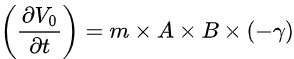 |
|  | Thermometer Resolution | B |  |  |
| **Repeatability** | Standard deviation referring to the calculated volume | A | 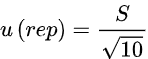 | 1 |
| **Meniscus reading** | Reference value | B | **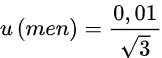** (15) | 1 |

**Table A.2. Sources that contribute to the expanded uncertainty of volumes transferred with Graduated Pipette.** Uncertainty factors: Type A – obtained in the test under repeatability conditions; Type B – obtained from available technical sources.
