# Supplementary material for: Development of a tool for measuring uncertainty of acute cytotoxicity results using the methodology of OECD Guidance Document 129
Source: NAM J. 2025 Apr 14;1:100021. doi: 10.1016/j.namjnl.2025.100021 (PMC13396913; doi:10.1016/j.namjnl.2025.100021)
Supplement: Supplementary file 2 [file mmc2.docx]

# Glossary

LD50 – Lethal Dose to 50% of animals;

IC50 – half-maximal Inhibitory Concentration to cell culture;

Cytotoxicity – capacity of a substance or process to damage or kill cells;

Measurement Uncertainty – a parameter that characterizes the dispersion of values that could be attributed to a measurement result;

Monte Carlo Simulation – a mathematical technique that predicts possible outcomes of an uncertain event.
